# A novel PPARɣ ligand, PPZ023, overcomes radioresistance via ER stress and cell death in human non-small-cell lung cancer cells

**DOI:** 10.1038/s12276-020-00511-9

**Published:** 2020-10-12

**Authors:** Tae Woo Kim, Da-Won Hong, Chang-Mo Kang, Sung Hee Hong

**Affiliations:** Division of Radiation Biomedical Research, Korea Institute of Radiological and Medical Sciences, Seoul, 139-706 Korea

**Keywords:** Cancer therapeutic resistance, Drug development

## Abstract

Peroxisome proliferator-activated receptor gamma (PPARɣ) agonists exert powerful anticancer effects by suppressing tumor growth. In this study, we developed PPZ023 (1-(2-(ethylthio)benzyl)-4-(2-methoxyphenyl)piperazine), a novel PPAR ligand candidate, and investigated the underlying signaling pathways in both non-small-cell lung cancer (NSCLC) and radio-resistant NSCLC cells. To identify whether PPZ023 has anticancer effects in NSCLC and radioresistant NSCLC cells, we performed WST-1, LDH, Western blot, and caspase-3 and -9 activity assays. Furthermore, we isolated exosomes from PPZ023-treated NSCLC cells and studied cell death signaling. PPZ023 reduces cell viability and increases LDH cytotoxicity and caspase-3 activity in NSCLC cells. PPZ023 induces cell death by generating reactive oxygen species (ROS) and triggering mitochondrial cytochrome c release. PPZ023 treatment causes cell death via the PERK–eIF2α–CHOP axis in both NSCLC cell lysates and exosomes, and PERK and CHOP knockdown significantly blocks ER stress-mediated apoptosis by reducing cleaved caspase-3. Interestingly, diphenyleneiodonium (DPI, a Nox inhibitor) inhibits PPZ023-induced cell death via ER stress, and PPARɣ knockdown inhibits PPZ023-induced ROS, ER stress, and cell death. Moreover, PPZ023, in combination with radiation, causes synergic cell death via exosomal ER stress in radioresistant NSCLC cells, indicating that PPZ023/radiation overcomes radioresistance. Taken together, our results suggest that PPZ023 is a powerful anticancer reagent for overcoming radioresistance.

## Cancer: Reversing resistance to radiotherapy

A novel small molecule drug candidate known as PPZ023 could be a powerful anti-cancer agent due to its ability to overcome the resistance of tumors to radiation therapy. Sung Hee Hong and colleagues at the Korea Institute of Radiological and Medical Sciences in Seoul, South Korea, investigated the effects of the molecule on lung cancer cells, including cells that that had acquired resistance to radiotherapy. PPZ023 induces the death of cancer cells by binding to a protein in a known signaling pathway, which generates damaging chemicals known as reactive oxygen species. The researchers identified additional molecular details of the anti-cancer activity. They found the radiotherapy resistance of cancer cells is reversed when PPZ023 promotes cell death via a pathway interfering with the folding of newly formed proteins in a cell structure called the endoplasmic reticulum.

## Introduction

Worldwide, lung cancer ranks highest in terms of both incidence and mortality^1^. Despite decades of research, systemic therapies fail to cure most lung cancers. Lung cancers comprise two major histological types: small-cell lung cancer (SCLC) and non-small-cell lung cancer (NSCLC; i.e., adenocarcinoma, squamous cell carcinoma, and large cell carcinoma)^2,3^. Despite of advances in chemotherapy, the prognosis of lung cancer remains poor. Chemotherapy, such as radiotherapy, is widely used in lung cancer patients, but chemoresistance is a major clinical obstacle^4^. Although radiotherapy is a powerful and effective treatment strategy in NSCLC, radioresistance also remains an obstacle^5^. An increasing number of studies have reported that radioresistance is related to various signaling pathways, including adaptive, DNA damage repair, adhesion, inflammation, and hypoxia pathways^6^. Radiation exposure frequently increases antioxidants, reactive oxygen species (ROS), and mitochondrial dysfunction, and this adaptive response is linked to radioadaptive resistance and pro-survival pathways^7^. ROS play a dual role in the cytotoxic effect of radiation therapy and the prosurvival adaptive response and cause radioresistance^8^.

PPARs play diverse roles in several aspects, such as lipid metabolism, immune disease, cell differentiation, and cell death^9^. Three PPARs- PPARα, PPARβ, and PPARɣ, regulate transcription by binding with peroxisome proliferator response element (PPRE) sequences and have different functions^10^. The PPARɣ ligand is an anti-diabetic drug and representative thiazolidinediones (TZDs) were the first drugs reported as PPARɣ agonists^11^. PPARɣ is expressed in diverse tumor types, such as lung cancer, and a therapeutic strategy using PPAR ligands is considered a potent and effective lung cancer therapy direction^12^. 15D-PGJ2, a putative natural PPARɣ ligand, exerts antitumor effects, including cell cycle arrest and caspase-dependent apoptosis, in choriocarcinoma cells^13^. Ciglitazone causes caspase-dependent cell death and ER stress in NSCLC cells, and the inhibition of ER stress blocks cell death^14^. Rosiglitazone inhibits cell proliferation through decreased Akt and mTOR phosphorylation and increased PTEN and AMPKα in NSCLC cells^15^. A recent report suggested that type 2 diabetes causes critical problems, such as the risk of diverse cancers, and mediates increased cancer incidence among people with diabetes^16^. The development of novel effective diabetic drugs provides new cancer therapy strategies, as metformin kills cancer cells and blocks tumor growth^17^.

Apoptosis is a highly regulated process that induces cell death and involves complex signaling cascades and stress responses^18,19^. Several studies have demonstrated that increased oxidative stress, DNA damage, endoplasmic reticulum (ER) stress, heat shock proteins, and mitochondrial stress serve as underlying mechanisms to induce cell death^20^. The endoplasmic reticulum (ER) is a multifunctional organelle responsible for lipid biosynthesis, vesicular trafficking, cellular calcium storage, and protein synthesis, folding, and export^21^. The ER provides a unique oxidizing environment for the folding of and disulfide bond formation in proteins before their transit to the Golgi compartment^22^. The unfolded protein response (UPR) is an adaptive signaling pathway designed to prevent the accumulation of misfolded proteins in the ER lumen and minimize the stress related to oxidative protein folding^23^. Four functionally distinct components of the UPR have been identified^24^. The first component involves the upregulation of ER chaperone proteins (GRP78/Bip and GRP94) to increase protein folding activity and prevent protein aggregation^25^. The second component consists of translational attenuation to reduce the load of new protein synthesis and prevent the further accumulation of misfolded proteins^26^. Translation inhibition is accomplished by PKR-like ER kinase (PERK), which phosphorylates and inhibits the subunit of eukaryotic initiation factor 2α (eIF2α) in response to ER stress^27^. The third component is an increase in the degradation of proteins misfolded in the ER by endoplasmic reticulum stress-associated degradation (ERAD), and the ERAD pathway signals to increase the expression of numerous genes involved in proteasome-mediated protein degradation^28^. The fourth element of the UPR involves the induction of apoptosis, which occurs when cytoprotective functions of the ER are overwhelmed^29^. Although the UPR is fundamentally a cytoprotective response, an excessive or prolonged UPR results in cell death, and various mechanisms have been suggested to play a role in ER stress-induced apoptosis^30^. These include the activation of c-Jun NH2-terminal kinase (JNK), the transcription factor C/EBP homologous protein (CHOP), and the BH3-only proteins PUMA, Noxa, and Bim^31^. Apoptotic cells showing compromised ER function, such as cells that are defective in the UPR or ER-associated protein degradation, are susceptible to ROS production. ER stress and oxidative stress are closely linked events, and ROS are an essential component of the events that lead to protein misfolding in the ER and ER stress-induced apoptosis^32–35^.

Our findings show that PPZ023 treatment triggers cell death via ROS and ER stress in NSCLC cells and that the inhibition of ROS or ER stress suppresses PPZ013-induced cell death.

## Materials and methods

### Reagents

Propidium iodide (PI) and antioxidants, including N-acetylcysteine (NAC), diphenyleneiodonium (DPI, a Nox inhibitor), and apocynin (Apo, an ROS inhibitor) were purchased from Sigma Chemical (St. Louis, MO USA). Ciglitazone and the selective PPARɣ antagonist, GW9662, were obtained from Cayman (Ann Arbor, MI). PPZ023 was synthesized in the Department of Chemistry at the College of National Science, Kongju National University (Gongju, Korea). PPZ023 was dissolved in dimethyl sulfoxide (DMSO) as a 1 mM stock solution. Annexin V-FITC kits were purchased from Biovision (Palo Alto, CA, USA).

### Cell culture

Human non-small-cell lung cancer cell lines (A549, H1299, and H460), a normal lung cell line (MRC5), and a mouse preadipocyte cell line (3T3L1) were purchased from the American Type Culture Collection (ATCC, Manassas, VA). A549, H460, MRC5, and 3T3L1 cells were cultured in DMEM (Welgene, Daegu, Korea) supplemented with 10% fetal bovine serum (FBS), 100 U/mL penicillin, and 100 mg/mL streptomycin (all from Welgene, Daegu, Korea) at 37 °C under a humidified 95/5%(v/v) mixture of air and CO_2_.

### Oil Red O staining

Ciglitazone (10 μM, 24 h), PPZ023 (25 μM, 24 h), and the PPAR-γ antagonist GW 9662 (10 μΜ, 24 h) were used to treat 3T3L1 preadipocytes. 3T3L1 cells were seeded into a 6-well plate at a density of 2 × 10^4^ cells per well in Dulbecco’s modified Eagle’s medium (DMEM) supplemented with 10% fetal bovine serum (FBS), 100 U/mL penicillin, and 100 μg/mL streptomycin. After 2 days, when 3T3L1 cells were at 80% confluence, the medium was changed to DMEM containing 10% FBS, 1 µM dexamethasone (Dex, Sigma-Aldrich), 0.5 mM 3-isobutyl-1-methyl-xanthine (IBMX, Sigma-Aldrich), and 5 µg/mL insulin (Sigma-Aldrich) for 48 h. After differentiation, the medium was changed to DMEM supplemented with 10% FBS and 5 µg/mL insulin twice every 48 h. On day 8, the ability of differentiated cells to accumulate intracellular lipids was assessed by Oil Red O staining. Cells were washed twice with PBS, chemically fixated using 3.7% formaldehyde solution, which was removed by rinsing in PBS twice, stained with Oil Red O working solution for 30 min at room temperature, and washed with deionized distilled water three times. Cells were then visualized by phase-contrast microscopy (Olympus, Tokyo, Japan), and images were obtained with a digital camera (Camedia C-5060, Japan). The Oil Red O stain was then eluted with isopropyl alcohol and its absorbance was measured at 510 nm.

### Determination of cell viability

Cell proliferation was assessed as a function of metabolic activity using WST-1 (4-[3-(4-iodophenyl)-2-(4-nitrophenyl)-2H-5-tetrazolio]-1,3-benzene disulfonate) assays (Roche Applied Science, Indianapolis, IN). The absorbance of each well was measured at 450 nm using an enzyme-linked immunosorbent assay reader (Bio-Rad Laboratories, Hercules, CA). For the WST-1 assay, human NSCLC cell lines (A549 and H460) and radioresistant NSCLC cell lines (A549R and H460R) were plated overnight into a 96-well plate. The cells were then treated with various concentrations of PPZ023 for various time periods.

### LDH assay

Human NSCLC cells were seeded into a 96-well plate with growth medium. To determine LDH (Thermo Scientific Pierce) activity in supernatants, 100 μL of the reaction mixture was added, and incubation for 30 min was performed in a dark room. LDH activity was determined by measuring the absorbance of the samples at 490 or 492 nm using the ELISA reader.

### Caspase-3 activity assay

Herein, 20 μg of total cellular protein was analyzed for caspase-3 activity using a PerkinElmer TruPoint caspase-3 assay system according to the manufacturer’s instructions. A549 and H460 cells were treated with Cig (30 μM; 24 h) and various doses of PPZ023 (0, 10, 25, and 50 μM; 24 h). Cell lysis buffer (50 μL) was then added, and cells were incubated on ice for 10 min. Samples were centrifuged at 10,000 g for 1 min and the protein concentration was quantified. Twenty micrograms of total cellular protein was added to and mixed with 2× reaction buffer (50 μL) and 4 mM DEVD-*p*NA substrate (5 μL). After incubation for 1 h at 37 °C, caspase-3 activity was analyzed at 405 nm using a spectrophotometer (Molecular Devices).

### Colony formation assay

A549, H460, and MRC5 cells were trypsinized, counted, and plated into 60-mm dishes at a density of 1000 cells/dish. Cells were treated as indicated and cultured for 10–12 days to allow colony formation. After incubation, colonies were fixed and stained with 1% methylene blue in 50% ethanol, and colonies comprising more than 100 cells were counted per plate. The survival fraction was calculated using the following formula: surviving fraction = (number of colonies formed/number of cells seeded) × plating efficiency of the control group.

### FACS

PPZ023-induced ROS during apoptosis were examined by monitoring the cells after staining with the cell permeant 2’7’-dichlorodihydrofluorescein diacetate (CM-H_2_DCFDA, Invitrogen). For annexin V/PI measurements, the cells were stained with the dye for 30 min using Annexin V-FITC kits (Biovision, Palo Alto, CA, USA), in accordance with the manufacturer’s instructions. The number of apoptotic cells was evaluated using a FACScan cytometer (Program Cell Quest, BD Biosciences).

### Ionizing radiation (IR)

IR exposure (2 Gy) was performed using ^137^Cs as the radiation source (Atomic Energy of Canada, Ltd., Mississauga, ON, Canada). IR-treated cells were used for experiments after IR exposure.

### Generation of radioresistant A549 and H460 cell lines

Cells were subjected to 2 Gy radiation daily for 3 months, except for weekends. Throughout the IR process and recovery time, cells were maintained at 40–70% confluence to ensure potential for exponential growth. After the second week of radiation exposure, the cells were maintained in DMEM containing 10% FBS and then washed and resupplemented daily for the first seven days and then every third day thereafter. Radio-selected radioresistance was verified by comparing the radiosensitivity of the radiation-selected cells with their respective parental cell lines using a colony survival assay.

### Transfection

Human NSCLC cells in a 6-well plate were transfected with double-stranded siRNAs (30 nmol/mL) against PERK (Santa Cruz), CHOP (Santa Cruz), and PPARɣ (Santa Cruz) and an shRNA against PPARɣ (Santa Cruz) for 24 h using Lipofectamine 2000 reagent (Invitrogen, Grand Island, NY) according to the manufacturer’s protocol. For the reporter assay, plasmids were transiently transfected using Lipofectamine 2000 (Invitrogen). To confirm the results of the luciferase reporter assay, a luciferase pGL3 vector (2 µg, Promega) and a PPRE-luciferase pGL3 vector (2 µg, Promega) were cotransfected with 0.2 g of CMV-β-GAL, a eukaryotic expression vector in which the *Escherichia coli* β-galactosidase (Lac Z) structural gene is under the transcriptional control of the CMV promoter. Luciferase reporter activity was assessed on a luminometer with a luciferase assay system (Promega, Madison, WI) according to the manufacturer’s protocol. Data for luciferase assays represent the mean ± SD of three independent experiments.

### Western blot analysis

Whole-cell extracts were obtained in RIPA lysis buffer (20 mM Tris-HCl, pH 7.5, 150 mM NaCl, 1 mM Na_2_EDTA, 1 mM EGTA, 1% NP-40, 1% sodium deoxycholate, 2.5 mM sodium pyrophosphate, 1 mM β-glycerophosphate, 1 mM Na_3_VO_4_, and 1 µg/ml leupeptin). Western blotting was performed using β-actin (Santa Cruz, 1:1000, sc-47778), CD63 (Abcam, 1:1000, ab118307), Nox4 (Proteintech, 1:1000, 14347-1-AP) and PPARɣ (Proteintech, 1:1000, 16643-1-AP) as well as cleaved caspase-3 (Cell Signaling, 1:1000, #9664), cleaved caspase-9 (Cell Signaling, 1:1000, #20750), cytochrome C (Cell Signaling, 1:1000, sc-11940), GRP78 (Cell Signaling, 1:1000, sc-3177), p-PERK (Thr980) (Cell Signaling, 1:1000, #3179), PERK (Cell Signaling, 1:1000, #5683), ATF4 (Cell Signaling, 1:1000, #11815), CHOP (Cell Signaling, 1:1000, #2895), eIF2ɑ (Cell Signaling, 1:1000, #5324), and p-eIF2ɑ (Ser51) (Cell Signaling, 1:1000, #3398). Protein samples were heated at 95 °C for 5 min and analyzed by SDS-PAGE. Immunoblot signals were developed by chemiluminescence with an enhanced ECL-plus substrate (Amersham Biosciences, Little Chalfont, Buckinghamshire, United Kingdom).

### Measurement of reactive oxygen species (ROS)

NSCLC cells were exposed to 25-μM PPZ023 for 8 h. ROS generation was measured after staining with 5-(and-6)-carboxy-2′,7′-dichlorodihydrofluorescein diacetate (DCF-DA; Molecular Probes), which interacts with ROS to form a fluorescent complex. DCF fluorescence was immediately measured by FACSCalibur flow cytometry (Becton Dickinson). Data were acquired and analyzed using BD CellQuest Pro software.

### Exosome isolation

Exosomes were obtained from the supernatant of DMSO- and PPZ023 (25 μM)-treated A549 and H460 cells according to the manufacturer’s protocol [Total Exosome Isolation Reagent (for cell culture media), Thermo Fisher Scientific]. The protein concentration was measured using the BCA method (Thermo Scientific). These protein loading samples (15 μg) were also quantified by Ponceau S staining and subjected to western blot analysis. Positive exosomes were identified using the exosome marker CD63.

### Statistical analysis

Data are expressed as the mean ± standard error (SE). Statistical analyses of the experimental data were performed using a two-sided Student’s *t*-test. *P* values < 0.05 were determined to indicate statistical significance.

## Results

### Identification of PPZ023 as a novel PPARɣ agonist in NSCLC and 3T3L1 cells

**Fig. 1:**
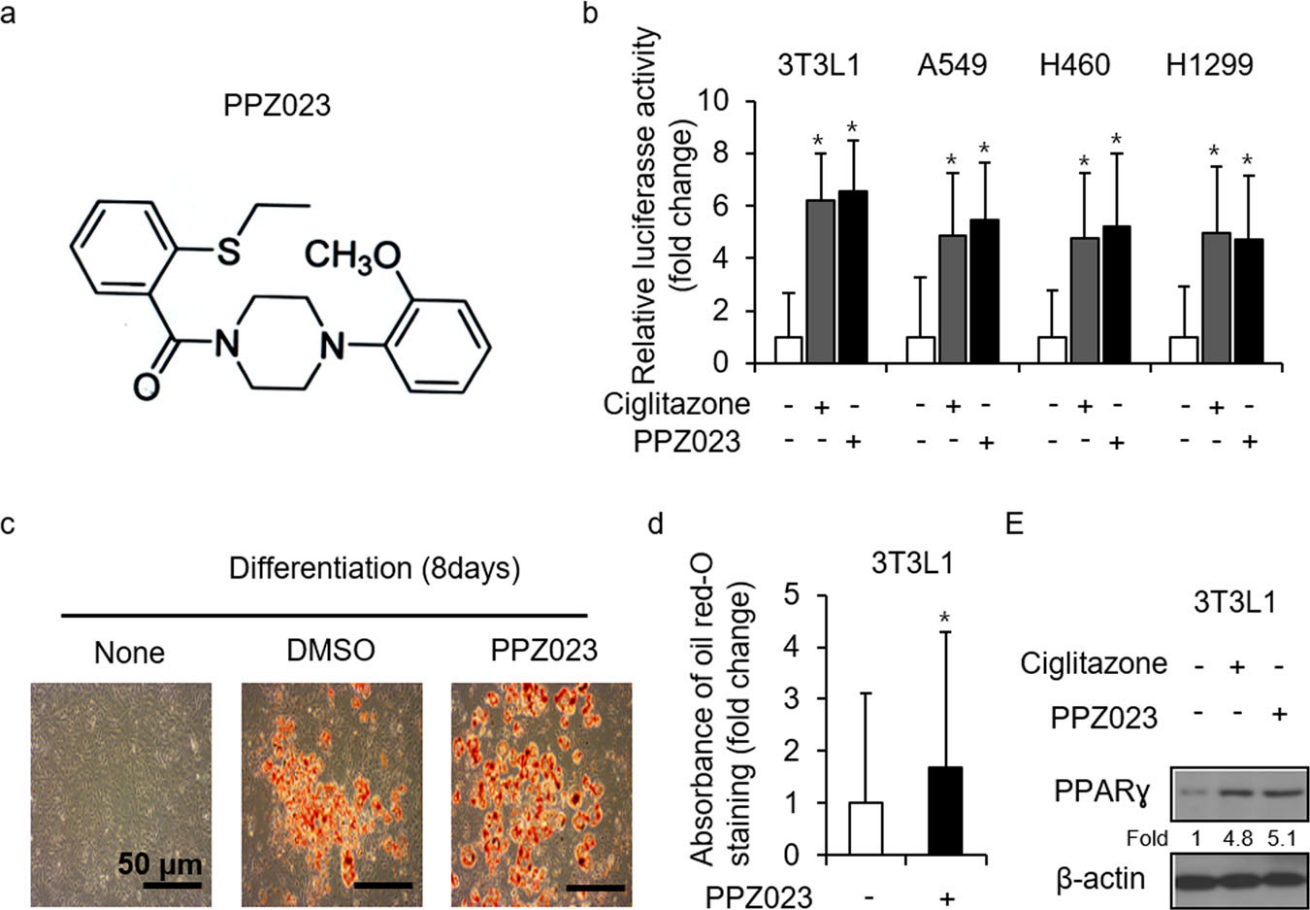
Adipocyte differentiation in PPZ023-treated 3T3L1 cells. **a** The chemical structures and molecular formulae of PPZ023. **b** 3T3L1, A549, H460, and H1299 cells were transiently transfected with 2 μg of the PPAR response element reporter gene (pGL3-PPRE vector) and the pGL3-PPRE vector and treated with PPZ023 or ciglitazone (Cig) at the indicated doses (PPZ023 25 μΜ; Cig, 10 μΜ); *p < 0.05. **c** PPZ023 (25 μΜ, 24 h)-mediated adipocyte differentiation was assessed by the presence of Oil Red O-stained droplets. Oil Red O-stained cells were detected using a light microscope by scoring cells from each dish at ×400 magnification. **d** Oil Red O quantification was performed by adding dye extraction solution to each well and measuring the absorbance at 510 nm. Values are shown as the mean ± SE of three replicates (*p < 0.05, **p < 0.01 vs. control; Student’s t-test). **e** The effect of PPZ023 (25 μΜ, 24 h) on 3T3L1 cells. Western blotting analyses were conducted to identify the activation of PPARɣ. β-Actin was used as the protein loading control.

We synthesized a novel potential PPARɣ ligand, PPZ023 (a1-(2-(ethylthio)benzyl)-4-(2-methoxyphenyl)piperazine), as shown in Fig. 1a. We performed PPRE-luciferase activity assays, and both PPZ023 and ciglitazone (Cig) caused enhanced luciferase activity in A549, H460, H1299, and 3T3L1 cells (Fig. 1b). To confirm PPZ023 as a PPARɣ ligand, we performed Oil Red O staining in preadipocyte mouse 3T3L1 cells. As shown in Fig. 1c, d, PPZ023 treatment increased adipocyte differentiation compared with control treatment. Western blot analysis revealed that PPZ023 and Cig also upregulated PPARɣ expression in 3T3L1 cells (Fig. 1e). These results suggest that PPZ023 is a potential novel PPARɣ ligand.

### PPZ023 causes cell death in NSCLC cells

**Fig. 2:**
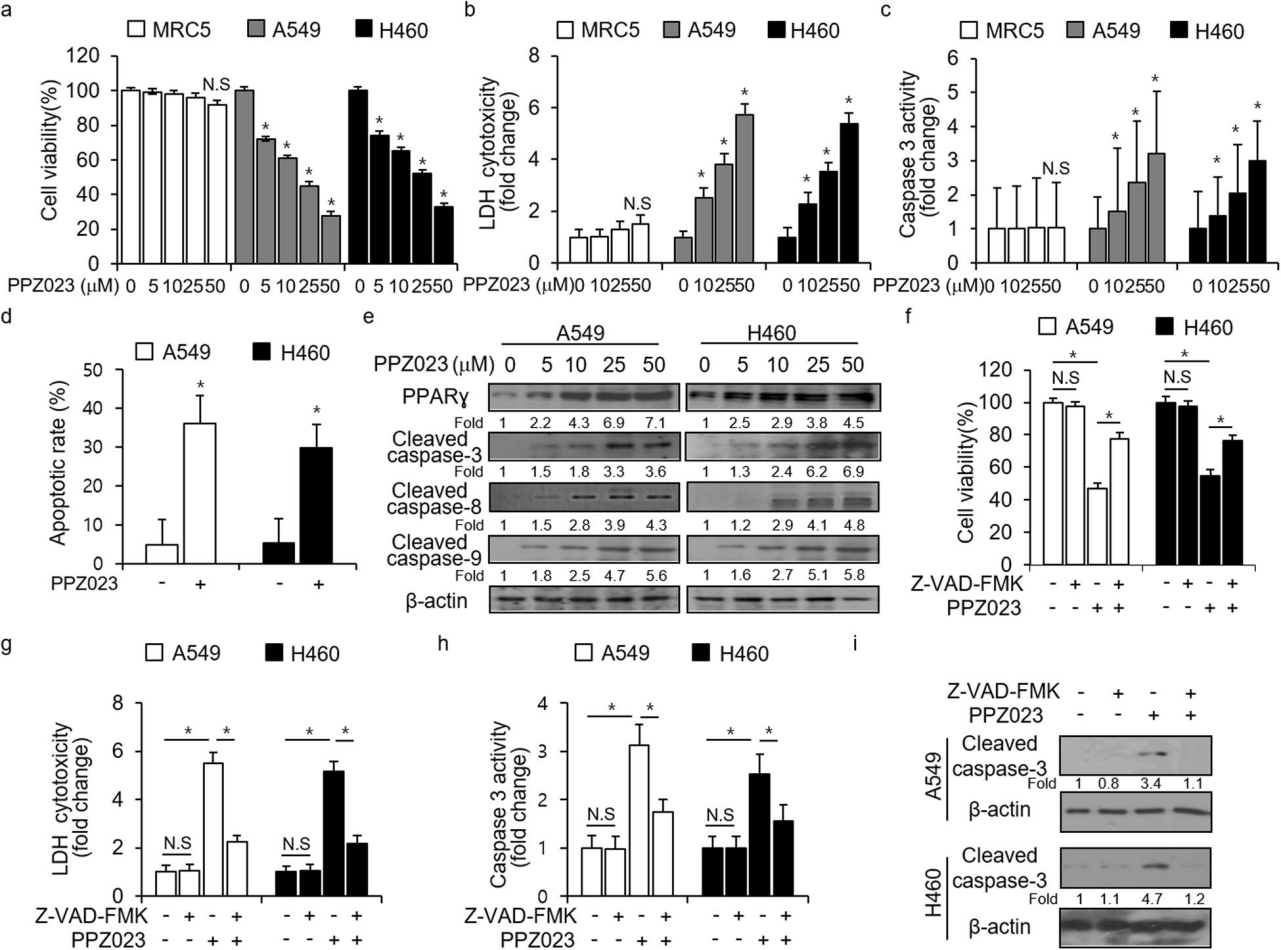
PPZ023 treatment induces caspase-dependent cell death in NSCLC cells. **a**–**c** MRC5, A549, and H460 cells were treated with PPZ023 at the indicated doses. WST-1 assays, LDH assays, and caspase-3 activity assays were performed under these condition; *p < 0.05. **d** Flow cytometry analysis of annexin-V-FITC and propidium iodide staining analyzed in PPZ023 (25 μM, 24 h)-treated A549 and H460 cells; *p < 0.05. **e** Western blot analysis of PPARɣ, cleaved caspase-3, and caspase-9 after treatment with PPZ023 (25 μM, 24 h) in A549 and H460 cells. β-Actin was used as the protein loading control. **f**–**i** A549 and H460 cells were treated with Z-VAD-FMK (50 μΜ) and PPZ023 (25 μM, 24 h). Cell viability, cell cytotoxicity, and caspase-3 activity were determined using WST-1, LDH, and caspase-3 activity assays; *p < 0.05. Protein samples were analyzed by western blotting of cleaved caspase-3. β-Actin was used as the protein loading control.

To test whether PPZ023 suppresses the growth of human NSCLC cell lines, H460 and A549 cells, as well as human normal lung cells, MRC5 cells, were treated with PPZ023 at different concentrations for 24 h, and cell viability, LDH cytotoxicity, and caspase-3 activity were analyzed. As shown in Fig. 2a–c, PPZ023 strongly inhibited the viability of NSCLC cells in a dose-dependent manner and increased LDH release, but had no effects on normal human lung fibroblast MRC5 cells. To examine the mechanism by which PPZ023 suppressed cell growth, A549 and H460 cells were treated with PPZ023 (25 μM, 8 h), and the annexin V assay was performed by flow cytometry (FACS). The results showed that compared with control cells, PPZ023-treated NSCLC cells contained increased numbers of apoptotic cells (Fig. 2d). Under different doses of PPZ023 treatment, Western blot analysis indicated that PPZ023 upregulated PPARɣ, cleaved caspase-3, cleaved caspase-8, and cleaved caspase-9 expression (Fig. 2e). To investigate whether PPZ023 regulates caspase-dependent cell death, we conducted pharmacological experiments using Z-VAD-FMK, a pan-caspase inhibitor. PPZ023/Z-VAD-FMK inhibited the decreased cell viability and increased LDH release and caspase-3 activity compared with PPZ023 alone in A549 and H460 cells (Fig. 2f–h). Western blot analysis also indicated that PPZ023/Z-VAD-FMK suppressed caspase-3 cleavage to a larger extent than PPZ023 alone in A549 and H460 cells (Fig. 2i). These data indicate that PPZ023 causes caspase-dependent cell death in NSCLC cells.

### PPZ023 causes cell death through ER stress in NSCLC cells

**Fig. 3:**
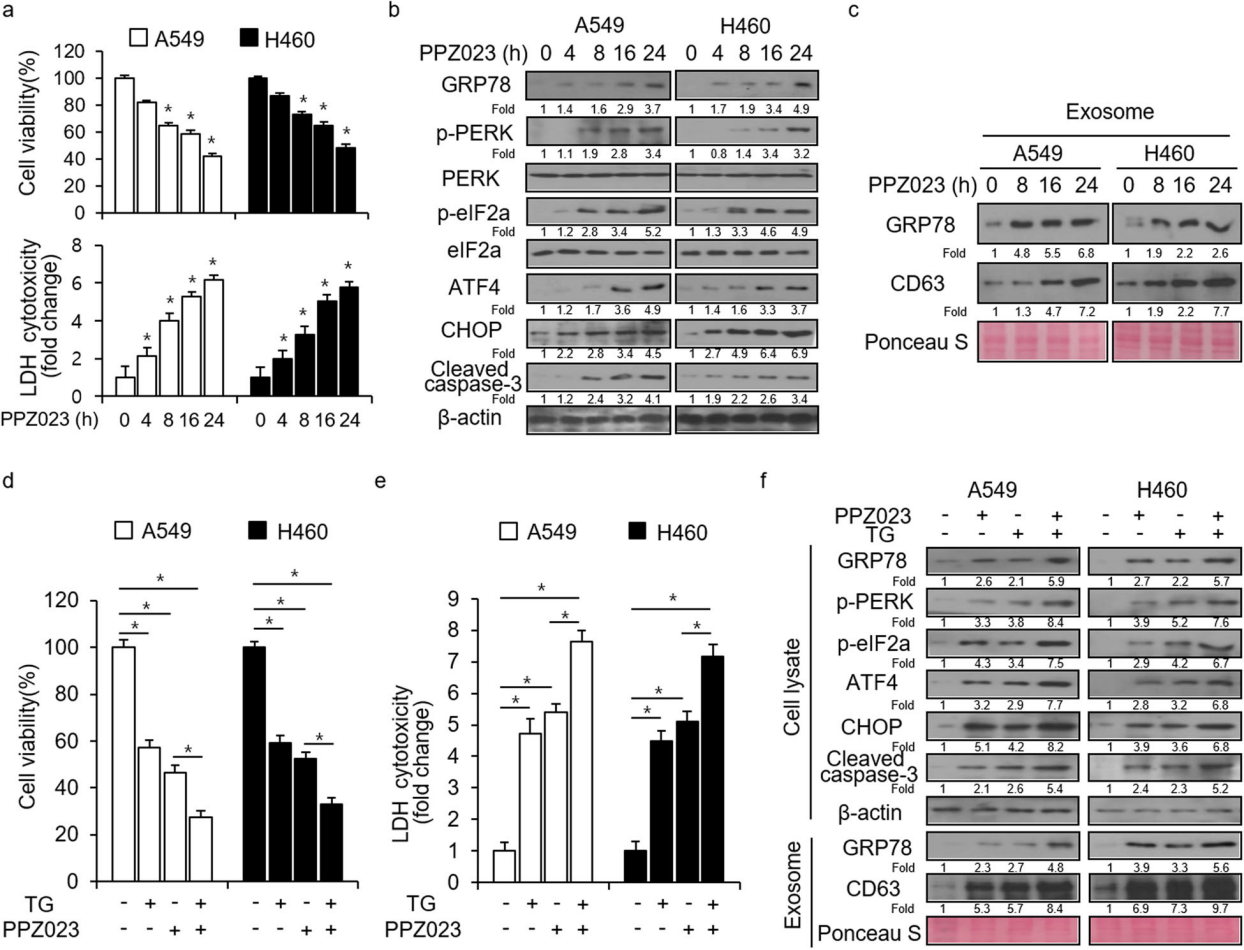
PPZ023 mediates ER stress and cell death through PERK signaling in NSCLC cells. **a** A549 and H460 cells were treated for various time periods (0, 4, 8, 16, and 24 h) with PPZ023 (25 μM), and WST-1 and LDH assays were performed under these conditions; *p < 0.05. **b**, **c** Protein samples isolated from cell lysates and exosomes were subjected to western blot analyses of GRP78, p-PERK, PERK, p-eIF2α, eIF2α, ATF4, CHOP, cleaved caspase-3, and CD63. β-Actin and Ponceau S stain were used as protein loading controls. **d**, **e** A549 and H460 cells were treated with TG (3 μΜ, 24 h) and PPZ023 (25 μΜ, 24 h). Cell viability and LDH release were determined using WST-1 and LDH assays, respectively; *p < 0.05. **f** Western blot analysis was performed to assess the protein expression of GRP78, p-PERK, p-eIF2α, ATF4, CHOP and cleaved caspase-3 and exosome expression of GRP78 and CD63. β-Actin was used as the protein loading control and Ponceau S stain was used as the exosome loading control.

To confirm whether PPZ023 blocks the growth of A549 and H460 cells in a time-dependent manner, the cells were treated with PPZ023 at the indicated times, and cell viability and LDH cytotoxicity were analyzed. As shown in Fig. 3a, PPZ023 strongly suppressed the viability of NSCLC cells and increased LDH release in a time-dependent manner. ER stress can be characterized by an increase in ER stress-associated molecules, including GRP78, p-PERK, p-eIF2α, and ATF6^36^. To determine whether PPZ023 causes cell death via ER stress in NSCLC cells, we investigated several crucial ER stress markers, including GRP78, p-PERK, PERK, p-eIF2α, eIF2α, ATF4, CHOP, and cleaved caspase-3. Our results revealed that PPZ023 exposure causes a time-dependent increase in the expression of GRP78, p-PERK, p-eIF2α, ATF4, CHOP, and cleaved caspase-3 in A549 and H460 cells (Fig. 3b). To probe whether PPZ023 causes ER stress in exosomes, we isolated exosomes from PPZ023-treated A549 and H460 cells and quantified them using Ponceau S staining. Western blot analysis suggested that PPZ023 upregulated the expression of the exosome markers CD63 and GRP78 in a time-dependent manner (Fig. 3c). These results show that PPZ023 can activate ER stress in cell lysates and exosomes from NSCLC cells. To investigate whether PPZ023 regulates cell death by activating ER stress, we performed pharmacological experiments using thapsigargin (TG), an ER stress inducer. TG and PPZ023 decreased cell viability and increased LDH cytotoxicity, respectively, in NSCLC cells (Fig. 3d, e). Furthermore, TG/PPZ023 decreased cell viability and increased LDH release compared with TG or PPZ023 alone (Fig. 3d, e). Western blot analysis indicated that TG and PPZ023 increased GRP78, p-PERK, p-eIF2α, ATF4, cleaved caspase-3, and CHOP expression in A549 and H460 cells, respectively, and that TG/PPZ023 resulted in higher GRP78, p-PERK, p-eIF2α, ATF4, cleaved caspase-3, and CHOP expression than TG or PPZ023 alone (Fig. 3f). Exosomes isolated from PPZ023-treated NSCLC cells were quantified by Ponceau S staining. Western blot analysis showed that TG and PPZ023 upregulated CD63 and GRP78 expression, respectively, and TG/PPZ023 resulted in higher CD63 and GRP78 expression than TG or PPZ023 alone (Fig. 3f). All these observations strongly suggest that PPZ023 induces cell death through exosomal ER stress in NSCLC cells.

### Inhibition of ER stress suppresses PPZ023-induced cell death in NSCLC cells

**Fig. 4:**
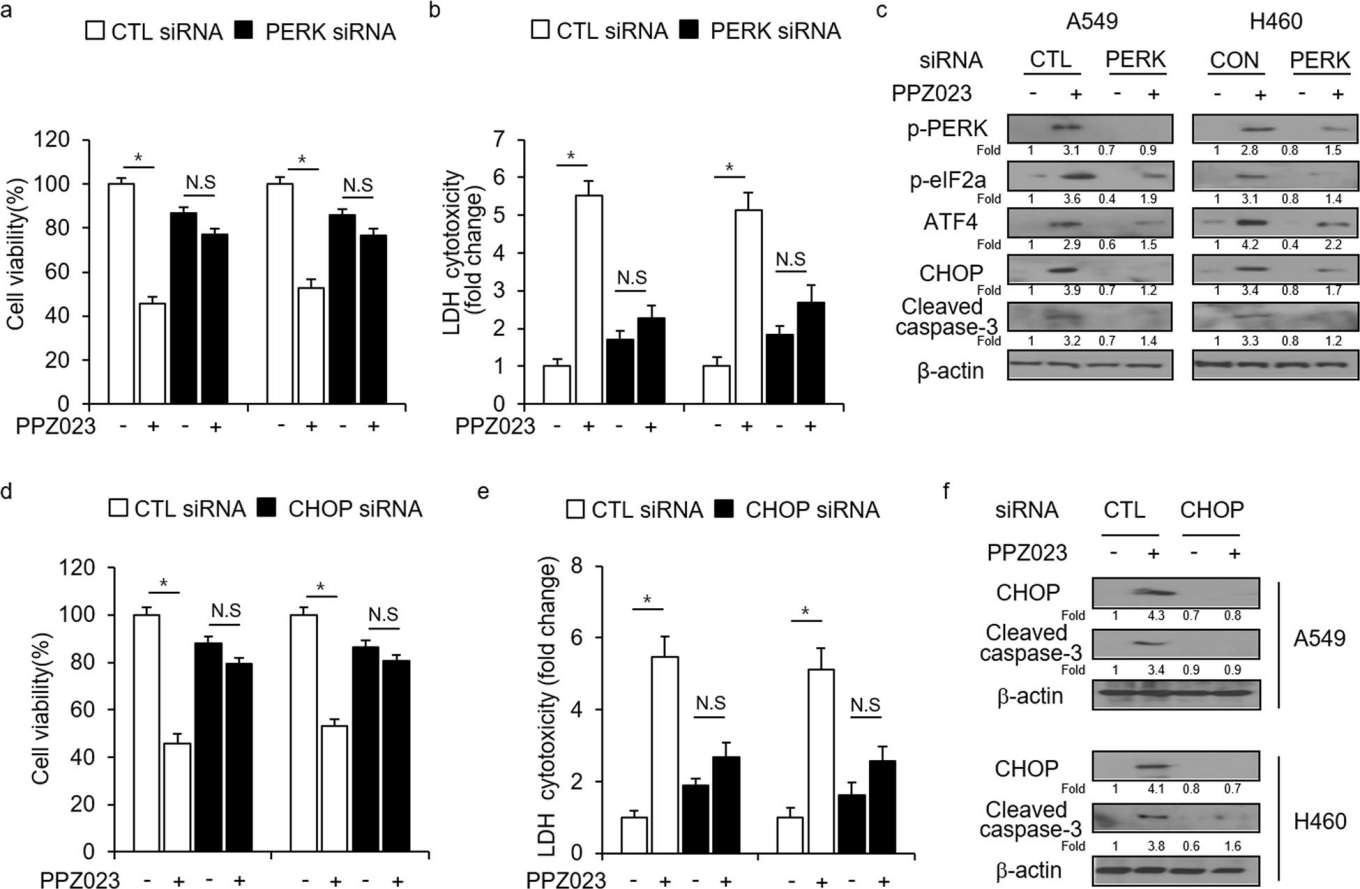
Suppression of PERK signaling inhibits PPZ023-induced cell death in NSCLC cells. **a**–**c** After A549 and H460 cells were transfected with PERK siRNAs, cell viability and LDH assays were performed, and Western blot analyses of p-PERK, p-eIF2ɑ, ATF4, CHOP, and cleaved caspase-3 levels were performed after PPZ023 (25 μΜ, 24 h) treatment; *p < 0.05. β-Actin was used as the protein loading control. **d**–**f** After A549 and H460 cells were transfected with CHOP siRNAs, cell viability and LDH assays were performed, and western blot analyses of CHOP and cleaved caspase-3 levels were performed after PPZ023 (25 μΜ, 24 h) treatment; *p < 0.05. β-Actin was used as the protein loading control.

To further investigate whether PPZ023 regulates cell death via ER stress in NSCLC cells, we knocked down PERK and CHOP. Our results indicated that PERK knockdown causes increased cell viability and decreased LDH cytotoxicity compared with control (CTL) knockdown in PPZ023-treated NSCLC cells (Fig. 4a, b). Western blot analysis suggested that PPZ023 but not PERK knockdown increased p-PERK, p-eIF2α, ATF4, cleaved caspase-3, and CHOP expression in CTL-transfected A549 and H460 cells (Fig. 4c). Additionally, we assessed the effects of CHOP knockdown on PPZ023-induced cell death. We found that CHOP knockdown also inhibited PPZ023-induced decreased cell viability and increased LDH release in A549 and H460 cells (Fig. 4d, e). Western blot analysis suggested that PPZ023 but not PERK knockdown increased caspase-3 cleavage and CHOP expression in CTL-transfected A549 and H460 cells (Fig. 4f). These results suggest that PPZ023 regulates cell death via ER stress in NSCLC cells.

**Fig. 5:**
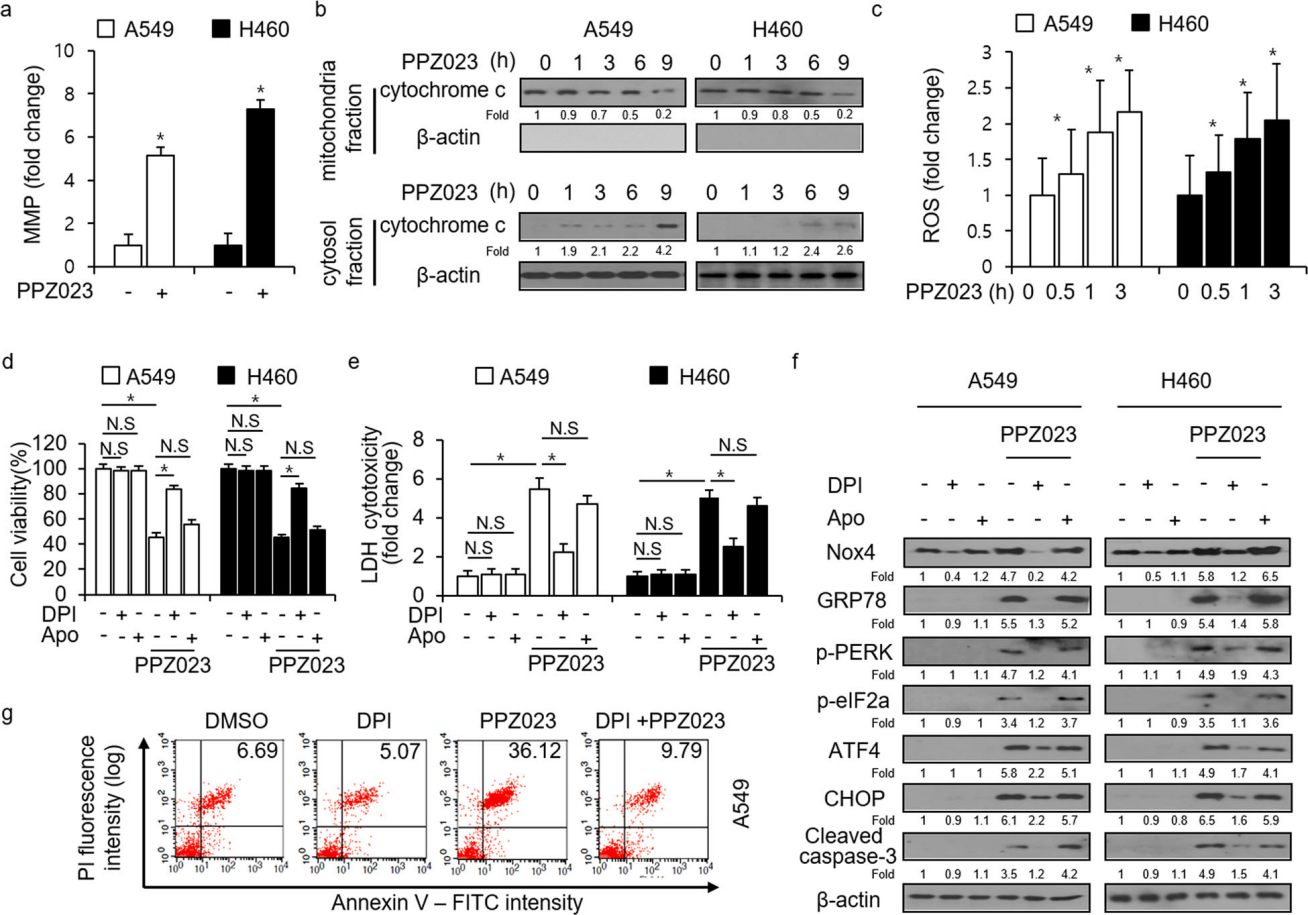
DPI inhibits PPZ023-mediated cell death in NSCLC cells. **a** PPZ023-induced ΔΨm loss was measured after staining A549 and H460 cells with DiOC_6_; *p < 0.05. **b** Release of cytochrome c from the cytosol and mitochondrial fraction in A549 and H460 cells treated with PPZ023 (25 μΜ) for the indicated times; this was detected by western blot analysis. **c** FACS data indicate changes in the fluorescence intensity of DCFDA dye in A549 and H460 cells treated with PPZ023 (25 μΜ) for the indicated times; *p < 0.05. **d**–**f** A549 and H460 cells were treated with DPI (1 μM, 24 h), Apo (100 μM, 24 h), and PPZ023 (25 μΜ, 24 h). Cell viability was determined using the WST-1 assay, and LDH cytotoxicity was measured using the LDH assay; *p < 0.05. Western blot analyses of GRP78, p-PERK, p-eIF2ɑ, ATF4, CHOP, and cleaved caspase-3 levels were performed on these samples. β-Actin was used as the protein loading control. **g** After A549 cells were treated with DPI, PPZ023, or DPI + PPZ023, annexin V FACS analyses were performed.

The loss of mitochondrial membrane potential (MMP) is considered a critical mediator of apoptosis^37,38^. To determine whether PPZ023-induced apoptosis is associated with the loss of MMP, we analyzed the effect of PPZ023 concentrations (25 μM) for 8 h on MMP by FACS analysis using DiOC_6_ staining. As shown in Fig. 5a, MMP was lost in A549 and H460 cells treated with PPZ023. To examine whether PPZ023-induced apoptosis occurred through a mitochondrial pathway, the release of cytochrome c from the mitochondria to the cytosol was analyzed. As shown in Fig. 5b, cytosolic cytochrome c levels in A549 and H460 cells increased after PPZ023 treatment at the indicated times, whereas those in the mitochondria concurrently decreased, indicating the release of mitochondrial cytochrome c. To determine whether PPZ023 contributes to ROS generation in NSCLC cells, we performed FACS using DCFDA staining in NSCLC cells, and ROS were induced by PPZ023 treatment at the indicated times (Fig. 5c). To further determine the mechanism by which PPZ023 regulates ROS, we identified whether ROS inhibitors, including DPI (an NAD(P)H oxidase inhibitor) and Apo (a p47phox inhibitor), block PPZ023-induced cell death via ER stress in NSCLC cells. As shown in Fig. 5d, e, DPI and Apo did not influence cell viability or LDH cytotoxicity in NSCLC cells, whereas PPZ023 and Apo/PPZ023 decreased cell viability and increased LDH release, respectively; however, DPI/PPZ023 dramatically inhibited PPZ023-mediated reduced cell viability and enhanced LDH release. To further determine whether ROS generation is involved in PPZ023-induced ER stress in NSCLC cells, we performed Western blot analysis. As shown in Fig. 5f, PPZ023 and PPZ023/Apo increased Nox4, GRP78, p-PERK, p-eIF2α, ATF4, cleaved caspase-3, and CHOP expression, whereas PPZ023/DPI decreased Nox4, GRP78, p-PERK, p-eIF2α, ATF4, cleaved caspase-3, and CHOP expression. We performed the annexin V assay using FACS in A549 cells, and DPI was dramatically inhibited in PPZ023-induced apoptotic cells (Fig. 5g). These data indicate that PPZ023 causes NADPH oxidase-derived ROS generation and increases Nox4 expression in NSCLC cells and induces cell death via ER stress.

### Inhibition of PPARɣ blocks PPZ023-induced cell death in NSCLC cells

**Fig. 6:**
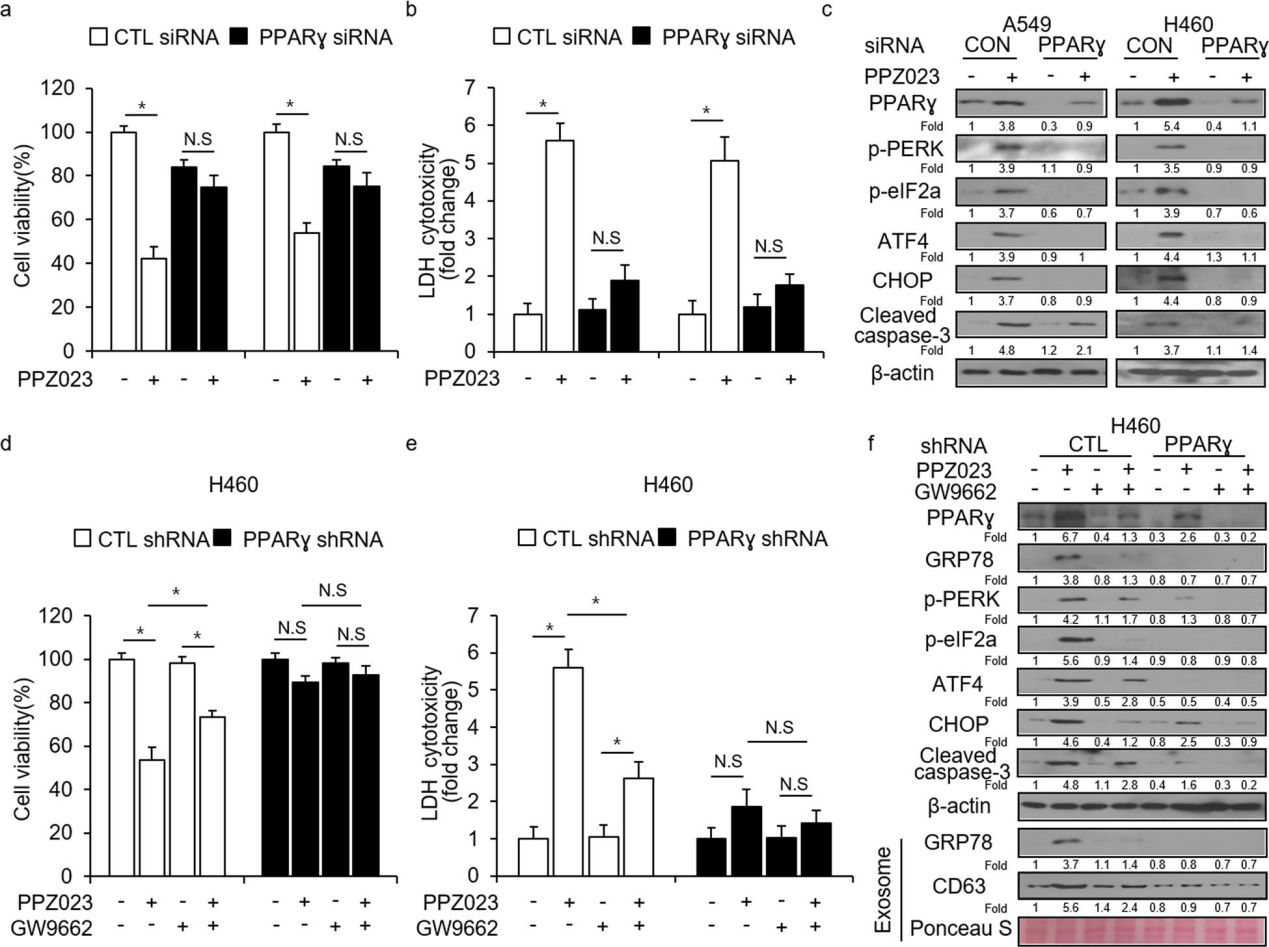
Suppression of PPARɣ inhibits PPZ023-induced cell death in NSCLC cells. **a**–**c** A549 and H460 cells were transfected with PPARɣ siRNAs and treated with PPZ023 (25 μΜ, 24 h). Next, cell viability and LDH assays were performed along with western blot analyses of PPARɣ, p-PERK, p-eIF2ɑ, ATF4, CHOP, and cleaved caspase-3; *p < 0.05. β-Actin was used as the protein loading control. **d**–**f** Stable PPARɣ-knockdown H460 cell lines were established after H460 cells were transfected with a PPARɣ shRNA. These cells were treated with PPZ023 (25 μΜ, 24 h), GW9662 (20 μM, 24 h), or PPZ023/GW9662, and cell viability and LDH assays were performed along with western blot analyses of PPARɣ, GRP78, p-PERK, p-eIF2ɑ, ATF4, CHOP, and cleaved caspase-3; *p < 0.05. β-Actin was used as the protein loading control, and Ponceau S stain was used as the exosome loading control.

PPARɣ ligands regulate cell death via a PPARɣ-dependent or -independent mechanism. To determine whether PPZ023 regulates cell death via PPARɣ activation, we conducted knockdown experiments using a PPARɣ siRNA in A549 and H460 cells, and then performed cell viability assays, LDH assays, and Western blot assays. Our data suggested that PPZ023 decreases cell viability and increases LDH release in CTL siRNA-transfected A549 and H460 cells; however, PPARɣ knockdown inhibits changes in cell viability and LDH cytotoxicity by PPZ023 (Fig. 6a, b). Western blot analysis indicated that PPZ023 increases PPARɣ, p-PERK, p-eIF2α, ATF4, cleaved caspase-3, and CHOP expression in CTL siRNA-transfected A549 and H460 cells; however, PPARɣ knockdown decreases the upregulation of these proteins (Fig. 6c). To further determine whether PPARɣ activation regulates PPZ023-mediated cell death in NSCLC cells, we transfected PPARɣ-specific shRNAs into H460 cells, and stable PPARɣ-knockdown cells were established with puromycin selection. We treated stable CTL- and PPARɣ-knockdown H460 cells with PPZ023, GW9662, and PPZ023/GW9662 and performed cell viability, LDH release, and Western blot assays. Our results indicated that PPZ023 decreases cell viability and increases LDH cytotoxicity in stable CTL-knockdown H460 cells, whereas GW9662 has no effect. PPZ023/GW9662 inhibited changes in cell viability and LDH production more effectively than PPZ023 alone; conversely, in stable PPARɣ-knockdown H460 cells, PPZ023, GW9662, and PPZ023/GW9662 had no effect on cell viability or LDH cytotoxicity (Fig. 6d, e). In stable CTL-knockdown H460 cells, Western blot analysis indicated that PPZ023 induced the expression of PPARɣ, GRP78, p-PERK, p-eIF2α, ATF4, CHOP, and cleaved caspase-3, whereas GW9662 and PPZ023/GW9662 blocked the expression of these proteins (Fig. 6f). In stable PPARɣ-knockdown H460 cells, PPZ023 did not affect the expression of the above markers (Fig. 6f). We isolated exosomes from cultured cells and quantified them using Ponceau S staining. Western blot analysis showed that PPZ023 increased CD63 and GRP78 expression in exosomes from stable CTL-knockdown H460 cells, whereas GW9662 and PPZ023/GW9662 did not (Fig. 6f). In exosomes from stable PPARɣ-knockdown H460 cells, PPZ023, GW9662 or PPZ023/GW9662 did not affect these proteins (Fig. 6f). To confirm whether PPZ023 regulates ROS release and cell death via Nox4 in NSCLC cells, we treated A549 and H460 cells with PPZ023 after knocking down Nox4 with siRNAs. PPZ023 increase cell viability and decreased LDH release in Nox4-knockdown cells compared with control (CTL)-knockdown cells (Supplementary Fig. 1a and b). Western blot analysis indicated that PPZ023 treatment suppresses CHOP expression in Nox4-knockdown cells compared with CTL-knockdown cells (Supplementary Fig. 1c). Together, our data indicate that PPARɣ activation by PPZ023 regulates ROS and the Nox4-induced cell death pathway in NSCLC cells.

### Both PPZ023 and radiation overcome radioresistance via ER stress

**Fig. 7:**
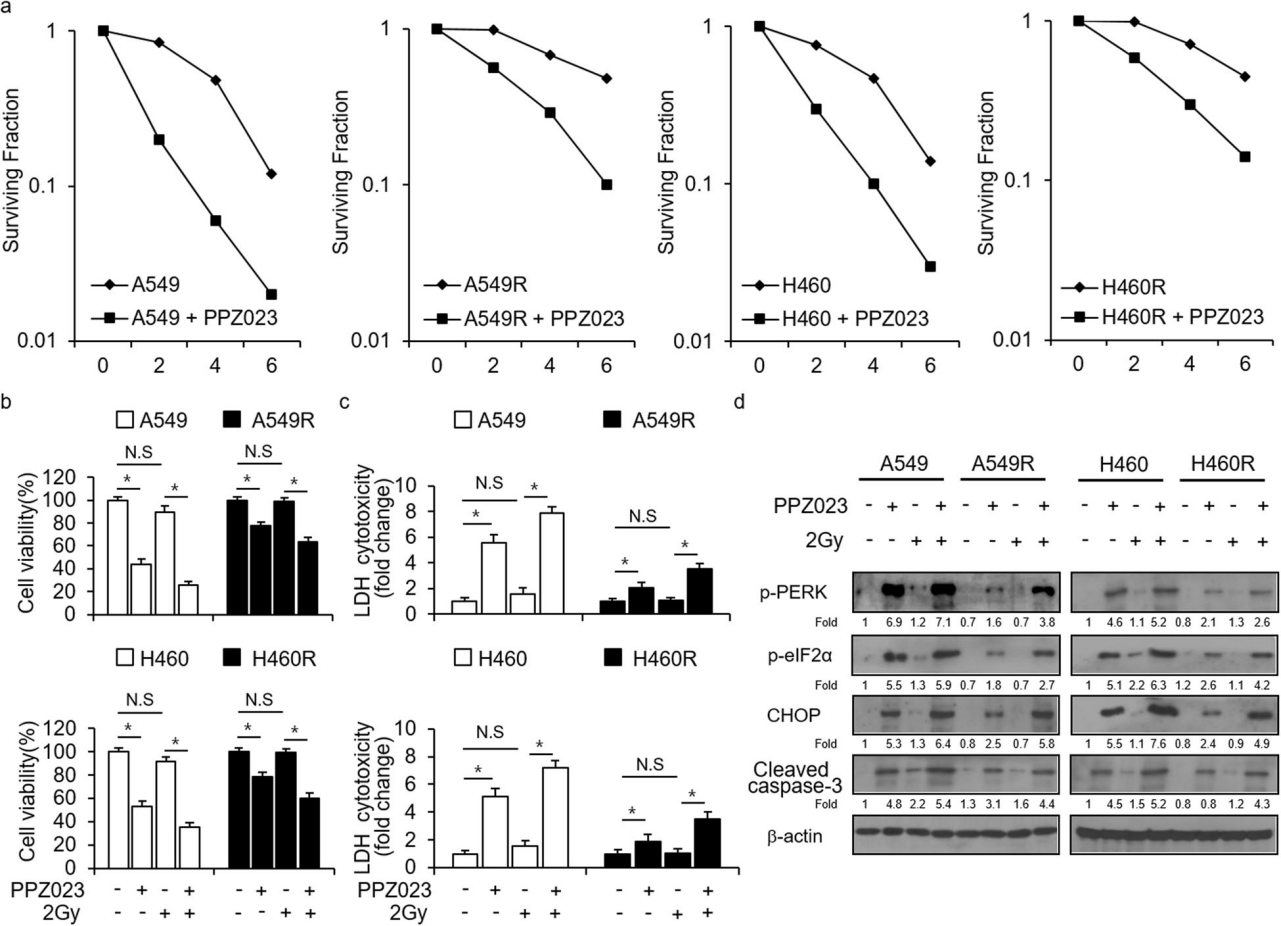
PPZ023/2 Gy induces cell death via ER stress in radioresistant NSCLC cells. **a** A clonogenic cell survival assay was conducted after PPZ023 (25 μΜ, 24 h) treatment and exposure to various radiation doses (2, 4, or 6 Gy). The survival fraction in A549, H460, A549R, and H460R cells was calculated using the surviving fraction formula; *p < 0.05. **b**–**d** A549, H460, A549R, and H460R cells were treated with PPZ023 (25 μΜ, 24 h) after exposure to 2 Gy radiation. Cell viability and LDH assays were performed along with western blot analyses of p-PERK, p-eIF2ɑ, CHOP, and cleaved caspase-3. β-Actin was used as the protein loading control.

Radiotherapy is a typical therapeutic strategy for the treatment of NSCLC; however, the obstacle of radioresistance is still unclear; thus, studies are needed to identify the mechanism underlying radioresistance^34,35^. To determine whether PPZ023 causes cell death and ER stress in radioresistant NSCLC cells (A549R and H460R cells), we performed the colony formation assays, WST-1 assays, LDH assays, and western blot analyses. Our data indicate that PPZ023 treatment decreases the fraction of surviving cells depending on the dose of radiation exposure (2, 4, and 6 Gy) in A549, A549R, H460, and H460R cells compared with CTL treatment (Fig. 7a). PPZ023 treatment caused lower surviving fractions in A549 and H460 cells than in A549R and H460R cells (Fig. 7a). PPZ023 decreased cell viability and increased LDH cytotoxicity in A549 and H460 cells compared with A549R and H460R cells, and PPZ023/2 Gy decreased cell viability and increased LDH release in A549 and H460 cells compared with A549R and H460R cells; however, 2 Gy alone had no effect (Fig. 7b, c). To investigate whether PPZ023/2 Gy confers cell death via ER stress in radioresistant NSCLC cells, we conducted western blot analysis. In A549 and H460 cells, PPZ023 increased p-PERK, p-eIF2α, CHOP, and cleaved caspase-3 expression, but 2 Gy did not (Fig. 7d). PPZ023/2 Gy induced increased p-PERK, p-eIF2α, CHOP, and cleaved caspase-3 expression compared with PPZ023 alone (Fig. 7d). However, in A549R and H460R cells, 2 Gy, PPZ023, and PPZ023/2 Gy had almost no effect on the expression of these proteins except for CHOP, which was weakly expressed (Fig. 7d). To identify the powerful cytotoxic effect of PPZ023, WST-1 and LDH assays were performed in A549R and H460R cells after treatment with PPZ023 and ciglitazone. Both PPZ023 and ciglitazone reduced cell viability and increased LDH release (Supplementary Fig. 2a and b). Western blot analyses indicated that PPZ023 and Cig increased CHOP levels in A549R and H460R cells (Supplementary Fig. 2c).

### PPARɣ inhibition suppresses PPZ023-induced ER stress in H460R cells

**Fig. 8:**
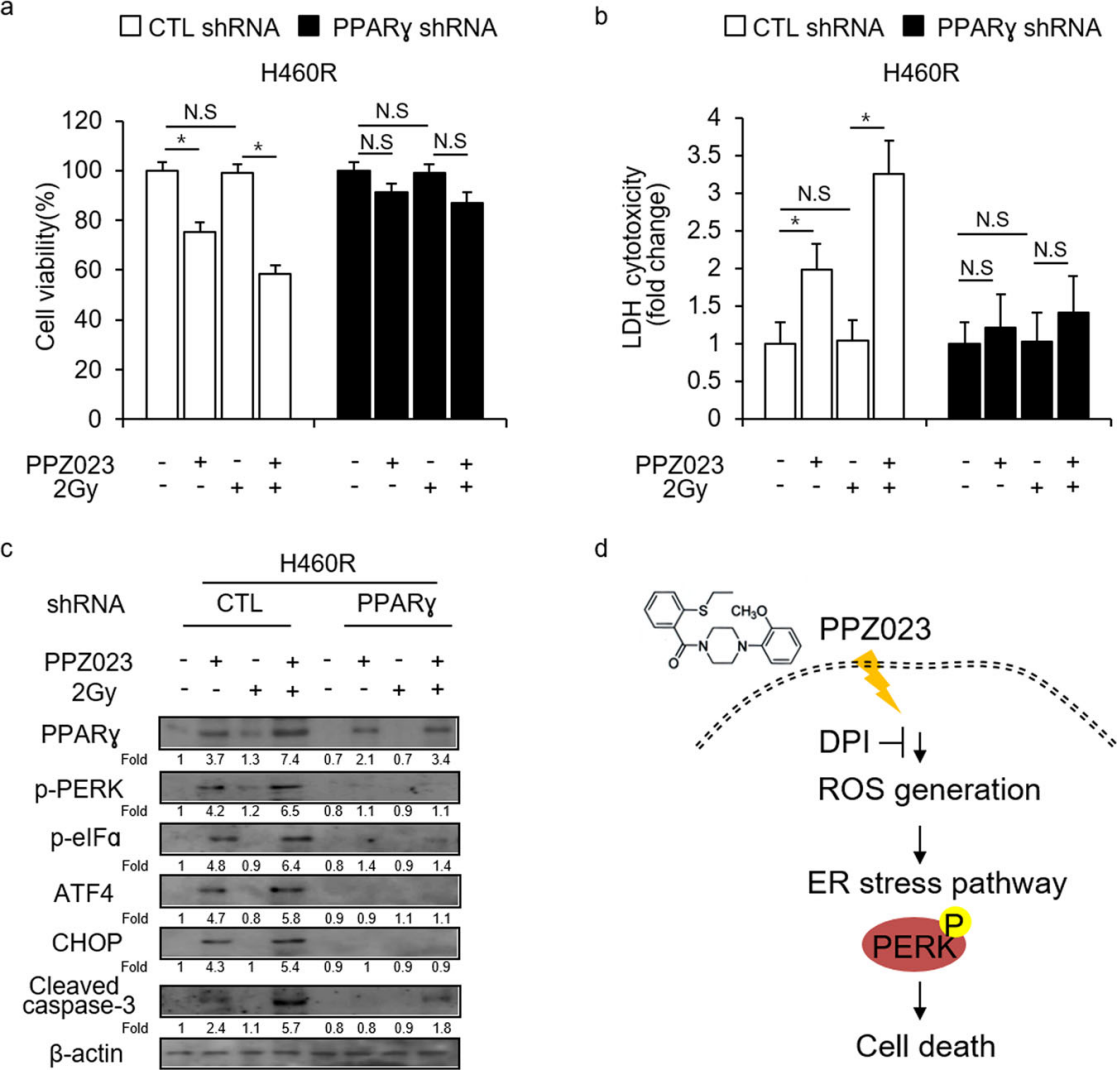
Inhibition of PPARɣ with a specific- shRNA suppresses PPZ023 + 2 Gy radiation-induced cell death in radioresistant H460R cells. **a**–**c** After H460R cells were transfected with the PPARɣ shRNA, stable PPARɣ-knockdown H460R cell lines were established. These cells were then treated with PPZ023 (25 μΜ, 24 h) after 2 Gy radiation exposure, and cell viability and LDH assays were performed along with western blot analyses of PPARɣ, p-PERK, p-eIF2ɑ, ATF4, CHOP, and cleaved caspase-3; *p < 0.05. β-Actin was used as the protein loading control. **d** Schematic representation of ER stress and cell death pathways stimulated by PPZ023 in NSCLC and radioresistant NSCLC cells.

To examine whether PPZ023/2 Gy induces ER stress and cell death in radioresistant H460R cells, we performed a transfection assay using the PPARɣ shRNA in H460R cells and established stable PPARɣ-knockdown cells using puromycin selection. Then, we treated the cells with PPZ023, 2 Gy, and PPZ023/2 Gy and performed WST-1 assays, LDH assays, and western blot analyses. In control shRNA-transfected H460R cells, PPZ023 decreased cell viability and increased LDH release; PPZ023/2 Gy decreased cell viability and increased LDH cytotoxicity; and 2 Gy alone had no effect. Conversely, in PPARɣ-knockdown cells, none of the three treatments (PPZ023, 2 Gy, and PPZ023/2 Gy) had any effect (Fig. 8a, b). Western blot analysis indicated that PPZ023 increases the expression of PPARɣ, p-PERK, p-eIFα, ATF4, CHOP, and cleaved caspase-3, but 2 Gy does not. Compared with PPZ023 alone, PPZ023/2 Gy increased the expression of PPARɣ, p-PERK, p-eIFα, ATF4, CHOP, and cleaved caspase-3 (Fig. 8c). In PPARɣ-knockdown H460R cells, only PPZ023 decreased the expression of PPARɣ, whereas 2 Gy and PPZ023/2 Gy had almost no effect. Taken together, our findings suggest that PPARɣ activation by PPZ023 causes cell death via ROS generation and ER stress, and plays a key role in PPZ023/radiation-induced radiosensitivity in radioresistant NSCLC cells (Fig. 8d).

## Discussion

In this study, we showed that PPZ023 inhibited the growth of NSCLC cells by inducing cell death via ROS, Nox4, and ER stress. Furthermore, PPZ023, in combination with 2 Gy, had synergistic anticancer effects, as indicated by cell death through ER stress in NSCLC and radioresistant NSCLC cells.

Apoptosis is a major biological process that causes cell death specifically via an intrinsic “suicide” mechanism, and its induction is believed to play a critical role in cancer progression^39^. In the present study, we found that PPZ023 increases caspase-3 activity and caspase-3 and caspase-9 cleavage in A549 and H460 cells. PPARɣ ligands induce apoptosis and cell cytotoxicity via oxidative stress in NSCLC cells and drug-resistant NSCLC cells^40^. Troglitazone, a PPARɣ ligand, inhibits tumor growth and induces apoptosis by downregulating Bcl-2 in breast cancer cells^41^. PPARɣ knockdown frequently inhibits PPARɣ-induced cell death, indicating a PPARɣ-dependent pathway^42^. Our results suggest that PPZ023 causes PPARɣ-dependent cell death via exosomal ER stress in A549 and H460 cells; however, PPARɣ knockdown suppresses cell death via ER stress in PPZ023-treated cell lysates and exosomes. Furthermore, PPZ023, in combination with 2 Gy of radiation, causes cell death in radioresistant H460R cells, but PPARɣ knockdown blocks PPZ023-induced cell death via ER stress. PPARɣ ligands activate unfolded protein responses, including protein synthesis, folding, and trafficking, in the ER, and excessive ER stress triggers cell death via ER stress transducers, including PERK, IRE1α, and ATF6^43^. The protein chaperone GRP78/Bip is the master regulator of these pathways^44^. With the accumulation of unfolded proteins, GRP78/Bip is released from IRE1 and permitted to dimerize via kinase and RNase activities to initiate XBP1 mRNA splicing to produce a potent transcriptional activator. Similarly, GRP78/Bip is released from ATF6 (ATF6 p90) and permits ATF6 transport to the Golgi compartment, where it is cleaved to yield a cytosolic fragment (ATF6 p50) that migrates to the nucleus to further activate the transcription of UPR-responsive genes^45^. Finally, GRP78/Bip release enables PERK dimerization and activation to phosphorylate eIF2α, and eIF2α phosphorylation induces the translation of ATF4 mRNA^45^. Our data indicate that PPZ023 causes cell death via the PERK–eIF2α–ATF4–CHOP pathway in NSCLC and radioresistant NSCLC cells and that the inhibition of PERK or CHOP inhibits PPZ023-induced cell death. CHOP is an ER stress-mediated cell death marker and a non-ER-localized multifunctional transcription factor that is induced by various adverse physiological and pharmacological conditions, including ER stress^46^. ER stress regulates CHOP transcription through two UPR pathways (PERK and IRE1α)^47^. The CHOP promoter contains an ERSE (ER stress-response element), which responds to agents that activate the mammalian UPR pathway, and a CEBP-ATF composite site, which has been shown to regulate CHOP transcription in response to various other stress conditions^47^. The activation of PERK leads to the phosphorylation of eIF2α and the synthesis of ATF4, and then, ATF4 together with C/EBPα binds to the composite site and transactivates the CHOP promoter^48^.

In the present work, we found that PERK activation and eIF2α phosphorylation are important for the PPZ023-mediated upregulation of CHOP in NSCLC cells and that the induction of CHOP causes cell death by PPZ023. TG (an ER stress inducer) and PPZ023 combination treatment increased ER stress markers, including GRP78, p-PERK, p-eIF2α, ATF4, cleaved caspase-3, and CHOP, in A549, H460, A549R, and H460 cells and GRP78 and CD63 in PPZ023-induced exosomes. PERK depletion decreased CHOP expression and caspase-3 cleavage in PPZ023-treated A549 and H460 cells, thus demonstrating the importance of this pathway. Additionally, CHOP knockdown suppressed PPZ023-mediated cell death by inhibiting cleaved caspase-3 expression.

Previous studies have suggested an association between protein folding and ROS generation that ultimately results in protein misfolding in the ER^49^. Several reports have revealed that ER stress occurs in response to oxidative stress during cell death triggered by various agents^50^. In this study, PPZ023 exposure caused a notable increase in ROS production and apoptosis in A549 and H460 cells. Moreover, treatment with DPI, an antioxidant, prevented PPZ023-induced ER stress and apoptosis by inhibiting ROS in A549 and H460 cells. Therefore, PPZ023 may induce cell death via oxidative stress and exosomal ER stress-induced signaling pathways in NSCLC and radioresistant NSCLC cells.

In conclusion, the findings of this study suggest that PPZ023 induces exosomal ER stress-induced cell death by mediating ROS in NSCLC and radioresistant NSCLC cells. In addition, it is suggested that a novel powerful anticancer drug, PPZ023, overcomes radioresistance after tumor radiotherapy.

## Supplementary information

Supplemental_information
