# Supplementary material for: A novel PPARɣ ligand, PPZ023, overcomes radioresistance via ER stress and cell death in human non-small-cell lung cancer cells
Source: Exp Mol Med. 2020 Oct 12;52(10):1730–43. doi: 10.1038/s12276-020-00511-9 (PMC8080717; doi:10.1038/s12276-020-00511-9)
Supplement: Supplementary file 1 — Supplemental_information [file 12276_2020_511_MOESM1_ESM.pdf]

A novel PPARɣ ligand, PPZ023, overcomes radio-resistance via ER stress and cell death in human non-small cell lung cancer cells

Tae Woo Kim, Da-Won Hong, Chang-Mo Kang and Sung Hee Hong

Supplementary Figure Legends

Figure S1.

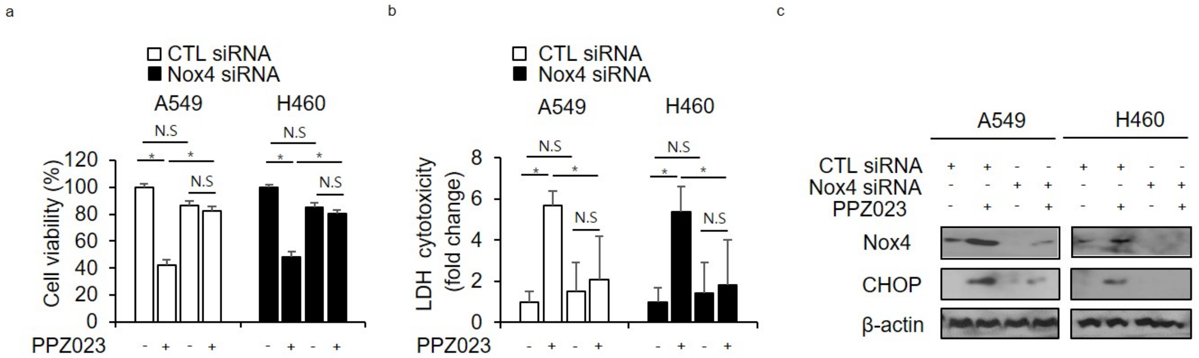

Figure S2.

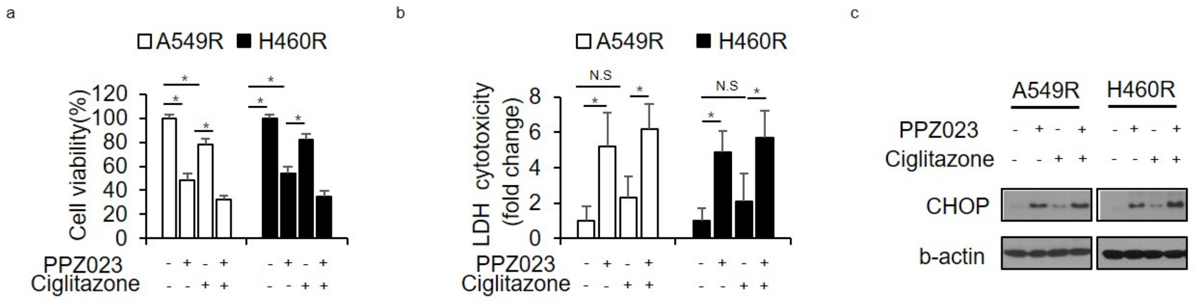

Figure S1.

Inhibition of Nox4 blocks PPZ023-mediated apoptotic cell death in NSCLC cells.

a-c A549 and H460 cells were transfected with Nox4 siRNAs and treated with PPZ023 (25 μM, 24 h). Cell viability assays, LDH assays, and Western blot analyses were performed to examine the expression levels of Nox4 and CHOP; *, $P<0.05$. β-actin was used as a protein loading control.

Figure S2.

Both PPZ023 and ciglitazone induces apoptotic cell death via ER stress in radiation-resistant NSCLC cells.

a-c A549R and H460R cells were treated with PPZ023 (25 μM, 24 h) and ciglitazone (10 μM, 24h). Cell viability was determined using a WST-1 assay, and LDH cytotoxicity was measured using an LDH assay; *, $P<0.05$. Western blot analyses were performed to examine CHOP levels; *, $P<0.05$. β-actin was used as the protein loading control.
